# Hyperphosphorylated Human Tau Accumulates at the Synapse, Localizing on Synaptic Mitochondrial Outer Membranes and Disrupting Respiration in a Mouse Model of Tauopathy

**DOI:** 10.3389/fnmol.2022.852368

**Published:** 2022-03-10

**Authors:** Andrew J. Trease, Joseph W. George, Nashanthea J. Roland, Eliezer Z. Lichter, Katy Emanuel, Steven Totusek, Howard S. Fox, Kelly L. Stauch

**Affiliations:** Stauch Laboratory, Department of Neurological Sciences, University of Nebraska Medical Center, Omaha, NE, United States

**Keywords:** aging, bioenergetics, proteomics, synaptic mitochondria, tau, phosphorylation, Alzheimer’s disease, tauopathy

## Abstract

Neurogenerative disorders, such as Alzheimer’s disease (AD), represent a growing public health challenge in aging societies. Tauopathies, a subset of neurodegenerative disorders that includes AD, are characterized by accumulation of fibrillar and hyperphosphorylated forms of microtubule-associated protein tau with coincident mitochondrial abnormalities and neuronal dysfunction. Although, *in vitro*, tau impairs axonal transport altering mitochondrial distribution, clear *in vivo* mechanisms associating tau and mitochondrial dysfunction remain obscure. Herein, we investigated the effects of human tau on brain mitochondria *in vivo* using transgenic htau mice at ages preceding and coinciding with onset of tauopathy. Subcellular proteomics combined with bioenergetic assessment revealed pathologic forms of tau preferentially associate with synaptic over non-synaptic mitochondria coinciding with changes in bioenergetics, reminiscent of an aged synaptic mitochondrial phenotype in wild-type mice. While mitochondrial content was unaltered, mitochondrial maximal respiration was impaired in synaptosomes from htau mice. Further, mitochondria-associated tau was determined to be outer membrane-associated using the trypsin protection assay and carbonate extraction. These findings reveal non-mutant human tau accumulation at the synapse has deleterious effects on mitochondria, which likely contributes to synaptic dysfunction observed in the context of tauopathy.

## Introduction

Altered synaptic bioenergetics and mitochondrial homeostasis are common features of several neurodegenerative disorders, including Alzheimer’s Disease (AD) (reviewed in Lin and Beal, 2006; Parihar and Brewer, 2007; Pathak et al., 2013; Norat et al., 2020). Whether observed mitochondrial aberrations are causal to, coincident with, or consequence of neurodegeneration is not well understood, however, a combination of these is likely (reviewed in Lin and Beal, 2006; Parihar and Brewer, 2007; Pathak et al., 2013; Norat et al., 2020). In AD, impaired respiratory chain function, adenosine triphosphate (ATP) production, Ca^2+^ buffering, neurotransmitter release, and altered redox balance have been reported to coincide with disease progression (Quintanilla et al., 2009; Rhein et al., 2009; Vos et al., 2010; Müller et al., 2011; Zündorf and Reiser, 2011; Pérez et al., 2018a). Furthermore, *in vitro* evidence suggests accumulation of the major histopathological hallmarks of AD—neurofibrillary tangles (NFTs) and amyloid plaques, comprised of microtubule-associated protein tau (Mapt) and amyloid-β, respectively—is associated with mitochondrial dysfunction and synaptic failure (Stelzmann et al., 1995; Rhein et al., 2009; Ittner et al., 2010; Vossel et al., 2010; Thompson and Vinters, 2012). Notably, synaptic deficits occur very early in AD and synapse loss is the most predictive of cognitive status (Thompson and Vinters, 2012). Although soluble oligomeric forms of amyloid-β are implicated in synapse loss early during AD progression, amyloid burden does not correlate well with synapse and neuron loss or severity of cognitive decline in AD (Serrano-Pozo et al., 2011; Nelson et al., 2012; Thompson and Vinters, 2012; Jung et al., 2016; Koss et al., 2018; Ower et al., 2018; Franzmeier et al., 2020). In contrast, NFTs, of which the majority of tau is aberrantly phosphorylated and misfolded, lead to synaptic alterations and correlate with neuronal loss and cognitive deficits in AD patients more readily than does amyloid burden (Lewis et al., 2000; Jose Metcalfe et al., 2010; Nelson et al., 2012; Thompson and Vinters, 2012). Furthermore, while pathogenic deposition of both tau and amyloid affect neuronal health and function, studies have suggested amyloid toxicity is dependent on tau (Roberson et al., 2007; Rhein et al., 2009; Ittner et al., 2010; Vossel et al., 2010). The mechanism by which tau over-expression alters synaptic function remains poorly understood; however, mutant and hyperphosphorylated forms of tau have been shown to impact the regulators of mitochondrial function and homeostasis, altering molecular transport, mitochondrial maintenance (i.e., fission/fusion), and bioenergetic functions, implicating a mitochondrial contribution to the observed synapse deficits (Vossel et al., 2010; Kopeikina et al., 2011; Llorens-Martín et al., 2011; Shahpasand et al., 2012).

Classical studies on the molecular basis of AD and other dementia related neurodegenerative diseases have primarily focused on the contributions of amyloid (reviewed in Hernandez et al., 2018). More recently, however, scientific and clinical interest in tau protein has begun to grow. Under normal physiological conditions tau protein interacts with microtubules (MTs) via MT-binding regions, promoting MT stability and fast axonal transport of molecular cargos in neurons (Kanaan et al., 2011; Llorens-Martín et al., 2011; Morris et al., 2011; Shahpasand et al., 2012; Rodríguez-Martín et al., 2013; Kadavath et al., 2015; Lacovich et al., 2017; Avila et al., 2019; Venkatramani and Panda, 2019), which are tightly regulated processes, most notably by post-translational modifications of tau (i.e., phosphorylation) (Grundke-Iqbal et al., 1986; Jose Metcalfe et al., 2010; Shahpasand et al., 2012; Rodríguez-Martín et al., 2013; Despres et al., 2017; Cieri et al., 2018). MT-dependent transport of mitochondria along the axon is critical to maintain local ATP production in distal neuronal compartments, like the synapse (Sheng and Cai, 2016). Despite the importance of tau to normal cellular trafficking, tau knock-out (KO) mice are both viable and lack overt pathology, perhaps due to compensation by other microtubule associated proteins (e.g., Map1A and Map1B) (Harada et al., 1994; Dawson et al., 2001). This is supported by a study that found tau and Map1B double KO mice suffer a lethal phenotype, dying by 4 weeks of age (Takei et al., 2000). Interestingly, primary neuronal cultures from tau KO mice display shortened axonal tracts, decreased microtubular density, and a reduction in the number of cross-bridging between adjacent microtubules as well as between microtubules and the cell membrane (Kanai et al., 1989; Liu et al., 1999; Takei et al., 2000). Notably, in *in vitro* systems, tau accumulation drives aberrant mitochondrial accumulation at the synapse (Zhang et al., 2012); and increased accumulation of tau protein in presynaptic regions impairs synaptic function (Tracy and Gan, 2018). In AD and associated tauopathies, tau is aberrantly hyperphosphorylated and accumulates in neurons, where excess tau promotes the formation of paired helical filaments (PHFs). In consequence, tau binding affinity for microtubules is reduced, leading to proteostasis and ultimately, neurodegeneration (Grundke-Iqbal et al., 1986; Andorfer et al., 2003; Despres et al., 2017). Evidence suggests that overexpressed and phosphorylated tau impairs axonal transport of organelles causing synapse starvation, depletion of ATP, and neuronal damage (Khatoon et al., 1994; Kuznetsov and Kuznetsov, 2015; Menkes-Caspi et al., 2015). In htau mice, tau redistribution from the axons into the cell bodies occurs by 3 months of age, accumulation of hyperphosphorylated tau begins by 6 months of age and increases further by 13 and 15 months of age, aggregated tau and PHFs are detectable by 9 months of age, additionally synaptic dysfunction has been demonstrated by electrophysiology (Andorfer et al., 2003; Polydoro et al., 2009). Similar to human AD patients the majority of tau pathology in htau mice is found in the neocortex and hippocampus (Andorfer et al., 2003; Polydoro et al., 2009) and exhibits phosphorylation of key serine residues (S202 and S396) as early as 3 months in these regions (Neddens et al., 2020).

In addition to disrupted trafficking, accumulation of cytosolic hyperphosphorylated tau alters mitochondrial maintenance, disrupting the balance of the fission/fusion processes as well as impairing clearance of aged, damaged, and dysfunctional mitochondria through mitophagy (Quintanilla et al., 2009; Reddy et al., 2011; Shahpasand et al., 2012; Kadavath et al., 2015; Hu et al., 2016; Pérez et al., 2018b; Cummins et al., 2019). In models overexpressing human tau (htau) mitochondrial networks are elongated in neurons, which promotes increased production of reactive oxygen species (ROS) (DuBoff et al., 2012; Li et al., 2016). In line with this, tau has been reported to interact with a large number of proteins, a disproportionate number of which are either mitochondrial residents or regulators (Liu et al., 2016). Notably, included in this population are the fission protein dynamin-related GTPase (Drp1) and the E3 ubiquitin-ligase Parkin, two proteins that contribute to mitochondrial maintenance (Manczak and Reddy, 2012; Mietelska-Porowska et al., 2014; Hu et al., 2016; Li et al., 2016; Cummins et al., 2019). Importantly, the interaction of tau with Drp1 is enhanced by tau phosphorylation and further reduces mitochondrial fission, promoting elongation of mitochondrial networks and consequently disrupting mitochondrial membrane potential (Manczak and Reddy, 2012). Although loss of mitochondrial membrane potential is a trigger of mitophagy, tau-Drp1 interactions may blunt this process due to reduced mitochondrial fragmentation (Shutt et al., 2011; Shirihai et al., 2015; Gao et al., 2021). Further exacerbating this, tau can sequester Parkin in the cytosol, preventing mitochondrial recruitment (Hu et al., 2016; Cummins et al., 2019). Together, the decreased fission and clearance of damaged mitochondria, lead to accumulation of dysfunctional mitochondria which in turn may contribute to enhanced ROS production and development of bioenergetic deficits (Lin and Beal, 2006; Barbagallo et al., 2015; Zhou et al., 2017). Beyond impacting mitochondrial maintenance, tau may also directly affect bioenergetics through modulation of respiratory complex expression and function (David et al., 2005; Li et al., 2016; Tracy and Gan, 2018). Indeed, htau accumulation reduced ATP production and complex I activity, *in vitro* (Li et al., 2016). Similar observations have been reported in mutant tau models as well, including the P301L mouse model (David et al., 2005). In addition to inhibited complex I activity, reduced expression of complex I and complex V are observed in mutant tau models, in concert with elevated ROS and mitochondrial depolarization (David et al., 2005). Despite this, studies investigating the impact of non-mutant tau in this context are limited.

In the present study we investigated the outcomes of expression of human non-mutant tau and age-dependent tau pathological changes on synaptic and non-synaptic brain mitochondria using bioenergetics assays, proteomic analysis, and biochemical fractionation experiments in htau mice. These mice express non-mutant human tau (all six isoforms) in the absence of murine tau and suffer progressive tau aggregation and cognitive deficits in conjunction with accumulation of NFTs (Andorfer et al., 2003; Polydoro et al., 2009). Here, in htau mice, elevated levels of tau protein were observed in synaptic terminals, coinciding with impaired synaptic bioenergetics, without concomitant alterations in synaptosomal mitochondrial content or overt mitochondrial DNA (mtDNA) aberrations. Interestingly, while maximal mitochondrial respiration was impaired in synaptosomes from htau mice, basal mitochondrial respiration and ATP production was not altered. Furthermore, there was a significant increase in synaptic mitochondrial associated tau protein at 8-months of age compared to 5-month-old animals in htau mice only. This correlated with both age-dependent levels of tau phosphorylation and alterations in the bioenergetics of synaptic mitochondria. Additionally, carbonate extraction and protease digestion revealed mitochondrially associated tau is associated with, but not inserted into the mitochondrial surface. We have identified the presence of multiple pathologic forms of tau associated with synaptic mitochondria, indicating a potentially direct role for tau in the impairment of synaptic homeostasis.

## Materials and Methods

### Reagents and Materials

#### Animals

Htau mice (B6.Cg-*Mapt^TM 1(EGFP)Klt^*Tg(MAPT)8cPdav/J, Stock# 005491; Jackson Laboratory (JAX), (Bar Harbor, ME, United States) express all six isoforms (including both 3R and 4R forms) of human tau in the absence of murine tau. The generation and characterization of the htau mice has been described previously (Andorfer et al., 2003). Mice used in this study were male and females, aged to 5- or 8-months. For each experimental protocol, transgenic mice were compared to age-matched controls (C57BL/6J mice, Stock# 000664; JAX). Animals were fed standard mouse chow and water *ad libitum* and housed in a controlled environment under a 12-h light/dark schedule. Experiments were performed in accordance with National Institutes of Health guidelines under a protocol approved by the University of Nebraska Medical Center Institutional Animal Care and Use Committee.

#### Reagents

All chemicals and reagents were purchased from Sigma Aldrich (St. Louis, MO, United States) unless noted below.

#### Antibodies

**FIGURE 1:**
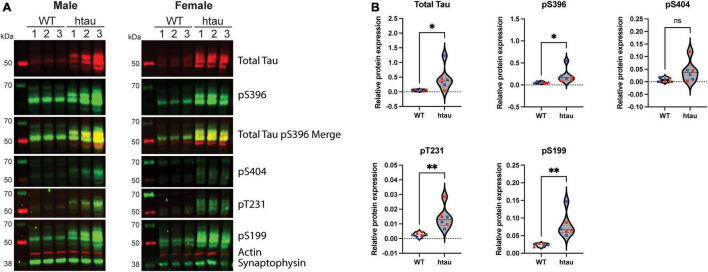
Phosphotau protein accumulates in synaptosomes of htau mice at 8-months of age. Synaptosomes were from 8-month-old WT and htau mice by discontinuous Percoll gradient, lysed, and evaluated by immunoblot for expression of total tau and phospho-tau. **(A)** Representative immunoblot results for male and female animals. **(B)** Densitometric quantitation is shown for males (blue) and females (red). Relative protein expression was determined by normalizing the indicated tau isotype to synaptophysin. Student’s T-tests were performed to determine statistical significance between WT and htau (*p < 0.05, ^**^p < 0.01; *n* = 6 per genotype, males and females were combined for analysis). ns, non-significant.

We used the following antibodies in this study: Tau46 (used in Figures 8, 9B; 1:1,000, #4019, Cell Signaling Technology, Danvers, MA, United States); Tau (used in Figures 1A, 9C, 1:1,000, #46687, Cell Signaling Technology, Danvers, MA United, States); CP13 Tau, PHF1 Tau, and MC1 Tau (all phospho-tau antibodies were used at 1:25 and obtained from Dr. Peter Davies, Feinstein Institutes for Medical Research); Tau pS404 (1:1,000, ab92676, AbCam, Cambridge, MA, United States); Tau pS396 (1:1,000, ab92676, AbCam, Cambridge, MA, United States); Tau pT231 (1:1,000, ab151559, Abcam, Cambridge, MA, United States); Tau pS199 (1:1,000, ab4749, AbCam, Cambridge, MA, United States); HSP60 (1:1000, #12165, Cell Signaling Technology, Danvers, MA, United States); Total OxPhos Rodent Antibody Cocktail (1:2,000, ab110413, Abcam, Cambridge, MA, United States); VDAC1 (1:2,000, #4661, Cell Signaling Technology, Danvers, MA, United States); SDHA (1:2,000, #11998, Cell Signaling Technology, Danvers, MA, United States); goat anti-mouse 680RD (P/N: 926-68070), goat anti-mouse 800CW (P/N: 926-32210), donkey anti-rabbit 680RD (P/N: 926-68073) and goat anti-rabbit 800CW (P/N: 926-32211) (1:20,000, Licor, Lincoln, NE, United States).

### Isolation of Nerve Terminals (Synaptosomes)

Mice were sacrificed by cervical dislocation and the brain was immediately removed, then rinsed with cold homogenization buffer (HB): 320 mM sucrose, 5 mM Tris, 1 mM EDTA (pH 7.4). The protocol was carried out on ice and all subsequent spins were done at 4°C. The brain was homogenized for 10 strokes with a Dounce homogenizer in HB containing cOmplete Mini, EDTA-free protease inhibitor cocktail (Roche Diagnostics, Indianapolis, IN, United States). The homogenate was then spun at 1,000 × *g* for 10 min and supernatant was collected. Following a second wash and spin at 1,000 × *g* for 10 min, the supernatants (approximately 4 mL) were pooled and diluted with 2 mL 3% Percoll in HB and layered on a discontinuous Percoll density gradient centrifugation as described previously (Stauch et al., 2014b). Following isolation, pelleted synaptosomes were resuspended in ionic media (20 mM HEPES, 10 mM D-glucose, 1.2 mM Na_2_HPO_4_, 1 mM MgCl_2_, 5 mM NaHCO_3_, 5 mM KCl, 140 mM NaCl, pH 7.4) for biochemical analysis or incubation buffer without glucose/pyruvate (IB^–^, 3.5 mM KCl, 120 mM NaCl, 1.3 mM CaCl_2_, 0.4 mM KH_2_PO_4_, 1.2 mM MgSO_4_, pH 7.4) for bioenergetic analysis. The isolated synaptosome protein concentrations were determined using the Pierce BCA Protein Assay Kit (Thermo Fisher Scientific, Waltham, MA, United States) and used immediately for ATP assay, bioenergetic analysis, flow cytometry, or lysed in 4% SDS/DTT and stored at −80°C. Protein quantification was performed using a Pierce 660 nm Protein Assay with bovine serum albumin standards with the addition of the ionic detergent compatibility reagent (Thermo Fisher Scientific, Waltham, MA, United States).

### Isolation of Brain Mitochondria

Mice were sacrificed by cervical dislocation and the brain was immediately removed, then rinsed with cold mitochondrial isolation buffer (MSHE): 70 mM sucrose, 210 mM mannitol, 5 mM HEPES, 1 mM EGTA and 0.5% (w/v) fatty acid free BSA (pH 7.2). The protocol was carried out on ice and all subsequent spins were done at 4°C. The brain was homogenized for 10 strokes with a Dounce homogenizer in MSHE containing cOmplete Mini, EDTA-free protease inhibitor cocktail (Roche Diagnostics, Indianapolis, IN, United States). The homogenate was clarified at 1,300 × *g* for 3 min and supernatant was collected. Following a second wash and clarification spin at 1,300 × *g* for 3 min, the supernatants were pooled and centrifuged at 21,000 × *g* for 10 min. Synaptic and non-synaptic mitochondria were isolated using Percoll density gradient centrifugation as described previously (Stauch et al., 2014b). Following isolation, pelleted mitochondria were resuspended in 1× mitochondrial assay solution (1× MAS; 70 mM sucrose, 220 mM mannitol, 10 mM KH_2_PO_4_, 5 mM MgCl_2_, 2 mM HEPES, 1 mM EGTA, pH 7.2). The isolated mitochondrial protein concentrations were determined using the Pierce BCA Protein Assay Kit (Thermo Fisher Scientific, Waltham, MA, United States) and used immediately for bioenergetic analysis or lysed in 4% SDS/DTT and stored at −80°C. Protein quantification was performed as described in the previous methods section for isolated synaptosoms.

### Bioenergetic Analysis of Isolated Brain Synaptic Terminals

Isolated synaptic terminals assessed functionally using the Seahorse Xfe96 analyzer based on the protocol of Choi (Choi et al., 2009) with minor alterations as described herein. Synaptosomes were isolated quantified by BCA (Pierce) and normalized to 1.5 mg/mL in IB supplemented with 4 mg/mL fatty acid free BSA (IB^+^). Fifteen microgram of total synaptosomes were plated per well on a poly-D-lysine coated culture plate (Agilent, Santa Clara, CA, United States) and centrifuged at 2,500 × *g* for 30 min at room-temp (RT). Well volume was brought to 175 μL with pre-warmed IB^+^ and then 5 μL of IB^–^ containing either pyruvate alone or pyruvate and glucose to a final concentration of 10 and 15 mM, respectively. For the Seahorse experiments, synaptosomes isolated from six retired breeder mice per strain (WT and htau) and sex at a range of 8-9 months of age were utilized. Each biological replicate (*n* = 5 or 6) had five technical replicate wells for each experimental condition. Tukey’s fence outlier test was used to exclude outlier biological replicates.

### ATP Assay

Synaptosomes isolated from 8-month-old WT or htau male and female retired breeder mice as described above were assessed for ATP production. Fifteen microgram of synaptosomes were plated on a 96-well plate in IB^+^ with three technical replicates per biological replicate. Wells were supplemented with substrate, either pyruvate alone or pyruvate plus glucose, with or without the addition of oligomycin. Samples were incubated in a non-CO_2_ incubator at 37°C for 30 min. Detection was accomplished using The Cell Titer Glo Luminescent Cell Viability (Promega G7571) following the manufacturer’s instructions. After incubation, the ATP assay solution was added to the wells at a 1:1 ratio. The plate was mixed on a shaker for 10 min at RT and luminescence was acquired using Synergy HTX BioTec plate reader. There were six (*n* = 6) biological replicates for each condition with three technical replicates per condition.

### Bioenergetic Analysis of Isolated Brain Mitochondria

The isolated synaptic and non-synaptic mitochondria were assessed functionally using the Seahorse XF24 analyzer based on the protocol of Rogers (Rogers et al., 2011) with minor alterations as described previously (Stauch et al., 2014a). For the Seahorse experiments, synaptic and non-synaptic mitochondria isolated from three mice per strain (WT and htau) at each of the two ages (5 and 8 months) were utilized for the coupling assay. Each biological replicate (*n* = 3) had three to four technical replicate wells for the experiment.

### Flow Cytometry

Synaptosomes were incubated with 50 nM MitoTracker Deep Red (MTDR, Invitrogen, Carlsbad, CA, United States) for 30 min at 37°C to stain mitochondria followed by incubation with 1 μg/μL FM 1-43 (Invitrogen, Waltham, MA, United States) for 1 min at room temperature to stain the synaptosomal membrane. These samples were run on the BD FACSAria II using a 488 nm laser for excitation and a 575/25 filter for emission (FM 1-43). MTDR was detected using a 633 nm laser for excitation and a 660/20 filter for emission. Data was analyzed using BD FACSDiva Software (Version 8.0.2).

### RT-qPCR

DNA was isolated from synaptosomes using the DNA micro kit (Qiagen, Germantown, MD, United States) per manufacturer protocol. A total of 20 ng of DNA was used as template for RT-qPCR analysis to determine mitochondrial DNA copy number was determined with the following primers and probe: 5′-CGTAGCCCAAACAA TTTCAT-3′ (forward), 5′-GTTTCTGCTAGGGTTGAGAT-3′ (reverse), 5′-/56-FAM/ACCCAAGAA/ZEN/CACATATGATTAC TTCTG C/31AbkFQ/-3′ (probe). The primers were designed to amplify the mouse mitochondrial genome (NCBI Ref Seq NC_005089.1) between 3,155 and 3,309. Samples were run on an Applied Biosystems StepOnePlus RT-qPCR machine and samples were subjected to an initial holding period of 2 min at 50°C followed by initial denaturation for 10 min at 95°C followed by 40 cycles of amplification and detection using a standard two-step protocol. Each cycle consisted of denaturation for 15 s at 95°C followed by combined annealing/extension for 1 min at 60°C, signal acquisition occurred in parallel with the extension. Relative fluorescence and threshold cycle time values were normalized per μg of synaptosome input (protein mass) for the DNA isolation.

### Nested PCR

Oligonucleotide primers were synthesized (Eurofins MWG Operon, Lancaster, PA, United States) to anneal to mtDNA segments flanking three direct repeats in the regions 8884-13357 (Wang et al., 1997). mtDNA was prepared from the synaptic mitochondrial fractions using the QIAamp DNA Micro Kit (Qiagen, Germantown, MD, United States; see above); the oligonucleotide outer primers, 5′-TAATTCAAGCCTACGTATTC-3′ (forward) and 5′-GGG ATGTTTTTAGGCTTAGG-3′ (reverse), and the oligonucleotide nested primers, 5′-CAAGTCCATGACCATTAACTGG-3′ (forward) and 5′-GATTTTATGGGTGTAATGCG-3′ (reverse) were used for the mtDNA deletion PCR reaction. As undeleted mtDNA controls in each sample, the mtDNA segment (471–670) encoding 12S rRNA was also amplified using 5′-GACAGCTAAGACCCAAACTG-3′ (forward) and 5′-TTAGCAAGAGATGGTGAGGT-3′ (reverse) primers. PCR conditions were initial denaturation at 98°C for 3 min, followed by 30 cycles of denaturation, annealing and extension at 98°C for 20 s, 55°C for 20 s and 72°C for 30 s, respectively, and final extension at 72°C for 2 min; 2× Q5 hot-start high fidelity master mix (New England Biolabs, Ipswich, MA, United States) was used. The outer primers were used for the first 30 cycles as described above with 20 ng mtDNA template, then 2 μL of the first reaction was transferred to the nested PCR for an additional 30 cycles. Resulting reactions were visualized by agarose gel electrophoresis.

### Mass Spectrometry-Based Proteomics

#### Protein Digestion

Mouse synaptic and non-synaptic mitochondrial protein sample aliquots (35 μg) used for data-independent acquisition mass spectrometry were digested with trypsin using filter aided sample preparation (Wiśniewski, 2017). The resultant peptides were desalted using Oasis mixed-mode weak cation exchanges cartridges (Waters, Milford, VA, United States), dehydrated with a Savant ISS 110 SpeedVac concentrator (Thermo Fisher Scientific, Waltham, MA, United States) and resuspended in 10 μL of 0.1% formic acid prior to quantification using a NanoDrop 2000 UV-vis spectrophotometer (Thermo Fisher Scientific, Waltham, MA, United States) in conjunction with the Scopes method for peptide quantification by absorbance at 205 nm (Scopes, 1974).

#### SWATH-MS Analysis

The samples of peptides (2 μg) from WT and htau mouse synaptic and non-synaptic mitochondrial lysates were analyzed in triplicate (three biological replicates per strain (WT and htau, *n* = 3) by nano-LC-MS/MS in SWATH-MS mode on the 5600 TripleTOF instrument (SCIEX) and targeted data extraction was performed as previously described (Stauch et al., 2014b,a). All fragment ion chromatograms were extracted and automatically integrated with PeakView software (Version 2.1, SCIEX, Framingham, MA, United States). For peptide identification, our published reference spectral library was used (Stauch et al., 2014b,a). This library was generated in ProteinPilot (Version 4.5, SCIEX) using the Paragon algorithm and the default settings. All searches were performed against the UniProt Mus Musculus Proteome UP000000589 containing 17,077 reviewed proteins (Swiss-Prot). Combined results yielded a library of 4,234 proteins identified with high confidence (greater than 99%) that passes the global false discovery rate (FDR) from fit analysis using a critical FDR of 1%. In accordance with previously published work (Stauch et al., 2014b,a), we selected five peptides and five transitions option for quantitative analysis and performed targeted data extraction for each peptide. For each peptide, the fragment ion chromatograms were extracted using the SWATH isolation window set to a width of 10 min and 50 ppm accuracy (Stauch et al., 2014b,a). To calibrate retention times, synthetic peptides (Biognosys AG, Schlieren, Switzerland) were spiked-in to the samples in accordance with the manufacturer’s protocol. Intensities were log_2_ transformed and quantile normalized across samples using the R language for statistical computing (Bolstad et al., 2003; R Core Team, 2021).

The mass spectrometry proteomics data have been deposited to the ProteomeXchange Consortium via the PRIDE (Vizcaíno et al., 2016) partner repository with the dataset identifier PXD030950.

#### Bioinformatics

The SWATH-MS data was uploaded to the MitoMiner 4.0 Web server^1^ (Smith and Robinson, 2019), a database of mammalian mitochondrial localization evidence, phenotypes, and diseases. Proteins deemed as mitochondrial were Gene Mito Evidence Mass-Spec Studies+, GFP+, Mass-Spec Experiments+, GO Annotation TRUE, IMPI known/predicted mitochondrial, and/or MitoCarta TRUE. Global pathway analysis was conducted in Ingenuity Pathway Analysis software (Krämer et al., 2014), using the Comparison Analysis tool and log_2_ transformed SWATH-MS expression values for proteins annotated as mitochondrial between WT and htau mice for each mitochondrial population. The Database for Annotation, Visualization and Integrated Discovery (DAVID) v6.8 was used to obtain Gene Ontology Biological Process terms enriched based on the lists of differentially expressed proteins (Huang et al., 2009b,a).

### Western Blot Analysis

#### Biochemical Isolation of Synaptic Mitochondrial Isolates

For mitochondrial membrane protein analysis 60 μg of Percoll-isolated mitochondria were resuspended in 1 mL of 0.1 M Na_2_CO_3_, pH 11.5, and incubated on ice for 30 min. After incubation samples were subjected to ultra-centrifugation at 100,000 × *g* for 20 min at 4°C. The insoluble membrane fraction (pellet) was solubilized by sonication and boiling at 95°C for 5 min in 30 μL solubilization buffer (SB; 100 mM Tris–HCl pH 7.4, 100 mM DTT, 4% w/v SDS). The soluble protein fraction (supernatant) was concentrated by centrifugation with an Omega NanoSep 30K NWCO spin-filter (Pall, Port Washington, NY, United States) at 14,000 × *g* until near dryness. Proteins were collected from spin-filters by pipette after the addition of 30 μL SB. The soluble protein fraction was then boiled at 95°C 5 min and sonicated briefly. Both fractions were quantified using the Pierce 660 nm Protein Assay (Pierce) with the IDCR (Thermo Fisher Scientific, Waltham, MA, United States). Equal masses of soluble and insoluble were analyzed by Western Blot.

#### Protease Treatment of Isolated Mitochondria

Synaptic and non-synaptic mitochondria from 8-month-old male htau mice were isolated by Percoll gradient as described above and final pellets were resuspended in 200 μL of 1× MAS and protein content was determined by Pierce 660 nm protein assay. Twenty microgram of total mitochondria were plated in each of seven wells in a round bottom 96 well plate on ice in a total volume of 15 μL. Protease digests were started by the addition 15 μL of Trypsin diluted in 1× MAS to yield the final working concentration indicated in Figure 9. Plates were incubated on ice for 30 min and mixed by gentle pipetting every 10 min. Once 30 min had passed, protease action was halted by the addition of 2 μL of 200 mM phenylmethylsulfonyl fluoride (PMSF), followed by gentle pipette mixing and an additional incubation of 10 min on ice. SDS-PAGE samples were prepared by the addition of 10 μL of 4× sample buffer (Licor, Lincoln, NE, United States), transfer to PCR strips tubes and boiling for 5 min at 95°C. Equal volumes of each sample were then resolved by SDS-PAGE, transferred to nitrocellulose, and immunoblotted as described below with the indicated primary antibodies. Quantification was carried out by densitometric measurements made in Light Studio (Licor).

#### Immunoblot Analysis

Isolated mitochondria or synaptosomes were lysed in 4% SDS and protein quantification was performed using a Pierce 660 nm Protein Assay with BSA standards with the addition of the IDCR (Thermo Fisher Scientific, Waltham, MA, United States). Fifteen microgram of lysate was resolved on Nu-PAGE Bolt 4–12% gradient polyacrylamide gels using the MES/SDS or MOPS/SDS buffer system (Life Technologies, Carlsbad, CA, United States), transferred to nitrocellulose using an iBlot2 instrument (Invitrogen, Waltham, MA, United States). Membranes were blocked with TBS/SuperBlock (Thermo Fisher Scientific, Waltham, MA, United States) for 30 min at RT, and then probed with the indicated antibodies at appropriate dilutions (see section “Antibodies”) in Tris buffered saline with 0.1% Tween-20 (TBS-T)/SuperBlock and incubated overnight at 4°C. Blots were washed 3 × 10 min with 1× TBS-T and then incubated with appropriate secondary antibodies (Licor) for 1 h at RT. Membranes were again washed 3 × 10 min with 1× TBS-T and imaged using an Odyssey imager (Licor) using appropriate channels. Quantification of immunoreactivity was achieved using Image Studio software (Licor).

### Statistical Analysis

Statistical analyses were performed in Prism 9 (GraphPad, San Diego, CA, United States) using ANOVA on the complete Seahorse assay data set with Sidak’s multiple comparisons *post hoc* testing and unpaired two-tailed *t*-tests for immunoblotting. The statistical tests performed are indicated in the figure legends. To uncover which proteins were differentially expressed (DE) in the SWATH-MS dataset, a ratio threshold (log_2_ htau/WT) was determined in addition to a *p*-value threshold. The ratio threshold was determined from the normal distribution fit using 1 standard deviation, where the absolute value of the z-score [normalized log_2_ ratios (htau/WT)] had to be superior to 1.0. To obtain *p*-values, the protein expression values for the biological replicates were analyzed using a Student’s *t*-test analysis, using *p* < 0.05 (uncorrected) for the threshold.

## Results

### Total and Phosphoforms of Tau Accumulate in Synaptosomes Isolated From Mice Expressing Non-mutant Human Tau

Histological and advanced imaging studies of tissue from AD patients have demonstrated that both total and abnormal forms of tau protein accumulate along axonal tracks as well as in synaptic terminals (Fein et al., 2008; Tai et al., 2012; Valotassiou et al., 2018; Vogel et al., 2020). Additionally, biochemical studies in rats and *Drosophila* have also reported accumulation of pathological forms of tau in pre-synaptic terminals (Jadhav et al., 2015; Zhou et al., 2017). In the htau mouse model classical AD histopathology is well established by 12-months of age, where the distribution of tau is similar to that observed in human brains (Polydoro et al., 2009); however, unlike in humans it is not yet clear if synaptic accumulation of pathological tau precedes cognitive impairment (Polydoro et al., 2009; Cho et al., 2021). With this in mind, we first sought to determine if tau accumulates in the synaptic terminals of htau mice. To address this, we isolated synaptosomes, which are derived from the termini of neurons, from 8-month-old wild-type (WT) and htau mice by discontinuous Percoll density gradient centrifugation and used immunoblotting to assess the levels of total and hyperphosphorylated tau (Figure 1). Compared to synaptosomes from WT animals, we observed increased expression of total tau and several phosphoforms (pS396, pS404, pT231, and pS199) in both male (Figure 1A left), and female (Figure 1A right) mice. Notably, pS396/pS404 phosphorylation is associated with the formation of PHF tau, and are the sites recognized by the PHF1 antibody (used in other assays) (Otvos et al., 1994). Concurrent immunohistochemical analysis of hippocampal sections confirmed characteristic distribution tau phosphorylated at positions relevant to AD [CP13 (pS202) and PHF1 (pS396/pS404); (Andorfer et al., 2003)] in the CA1 and CA3 in 8-month-old htau animals (Supplementary Figure 1). These findings suggest synaptic tau accumulation precedes cognitive impairment reported at 12 months in htau mice (Polydoro et al., 2009).

### Tau Accumulation Impairs Mitochondrial Maximal Respiration in Synaptosomes Isolated From Htau Mice

**FIGURE 2:**
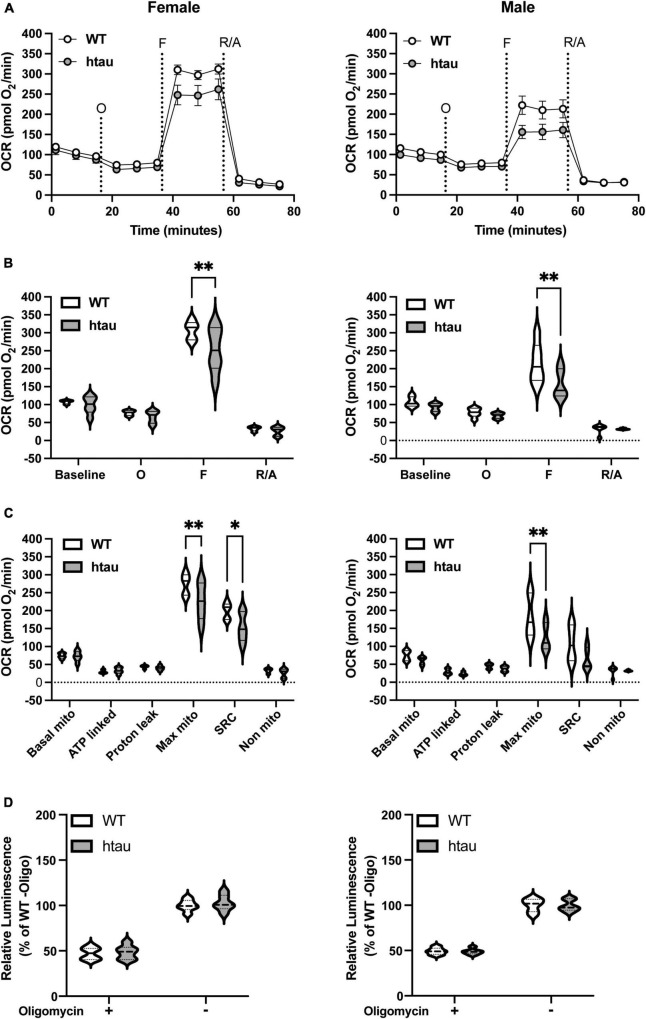
Mitochondrial bioenergetics are impaired in synaptosomes from htau mice. Synaptosomes were isolated from male and female WT and htau mice at 8-month of age and bioenergetics were assessed using the Seahorse MitoStress Test. Oxygen consumption rates **(A)** line graph and **(B)** bar plot. **(C)** Quantification of respiration profiles for basal, ATP-linked, proton leak, maximal, spare, and non-mitochondrial respiration. **(D)** Quantification of ATP production assay under identical substrate conditions with or without the addition of oligomycin. Tukey’s fence outlier test was used to exclude outlier biological replicates. Two-way ANOVA with Sidak’s multiple comparisons test was used to determine statistical significance (*p < 0.05, ^**^p < 0.01; WT male *n* = 6, htau male *n* = 5, WT female *n* = 5, htau female *n* = 5; with 5 technical replicate wells per biological replicate).

Synaptic transmission is a highly energy dependent process, requiring large amounts of localized ATP (Harris et al., 2012; Pathak et al., 2015). Additionally, bioenergetic deficits are common to several neurological disorders including AD (Lin and Beal, 2006; Parihar and Brewer, 2007; Rhein et al., 2009; Cheng et al., 2010; Harris et al., 2012; Pathak et al., 2013). With the high energetic demands of synaptic transmission and our observed increased tau expression in synaptic terminals of htau mice we sought to determine if tau accumulation alters synaptic bioenergetics. To test this, we used two assays, the Seahorse MitoStress Test (Figures 2A–C) and an ATP production assay (Figure 2D), supplying pyruvate and glucose as substrates. To better correlate the results of both assays, samples from the same animals were used in parallel. Seahorse analysis revealed an impairment in both mitochondrial maximal and spare respiratory capacities in synaptosomes of female htau mice compared to WT at 8 months of age. Like females, we observed a significant decrease in maximal respiration in htau males compared to WT, and a trending albeit non-significant impairment in spare respiratory capacity (*p* = 0.083). Notably, oxygen consumption rates (OCR), when mitochondrial ATP synthase (complex V, ATP-linked respiration) was inhibited by the addition of oligomycin, were comparable between WT and htau animals (Figure 2C). In agreement with this, we observed comparable ATP production in WT and htau animals in the presence and absence of oligomycin (Figure 2D), regardless of sex. Although, we did not directly measure glycolysis, it likely contributed to measured ATP production from the presence of both glucose and pyruvate as substrate. Taken together, our results suggest that the accumulation of tau impairs synaptic mitochondrial maximal respiration without impairing basal mitochondrial respiration or basal ATP production. This suggests a limited capacity of the synaptosomes to adjust mitochondrial oxygen consumption in response to energy demands.

### Synaptic (But Not Non-synaptic) Mitochondria Isolated From Htau Mice Exhibit Age-Associated Bioenergetic Alterations

**FIGURE 3:**
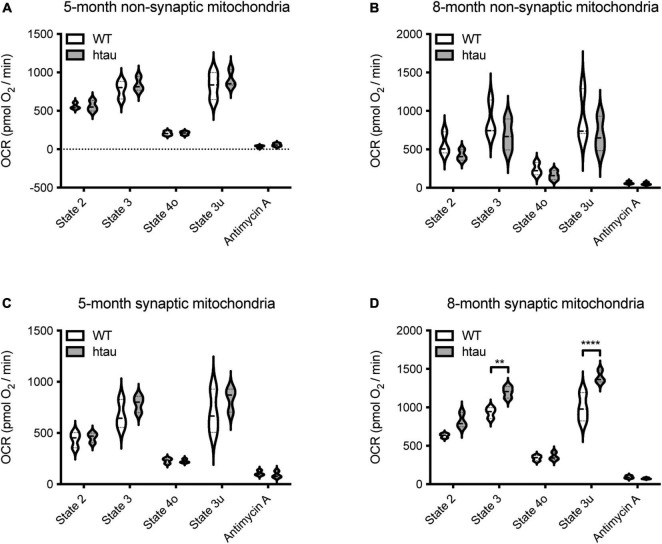
Tau expression alters synaptic mitochondrial respiration. Synaptic and non-synaptic mitochondria were isolated by discontinuous Percoll density gradient centrifugation from 5- and 8-month-old WT and htau mice and complex II-driven mitochondrial respiration **(A–D)** was assessed using a Seahorse XFe24 Analyzer with the coupling assay. Seahorse quantifications were normalized to protein content on a per well basis. Statistical significance was determined by 2-way ANOVA with Sidak’s multiple comparisons test (**p < 0.001, ****p < 0.0001; *n* = 3, with 3 or 4 technical replicate wells).

To determine if mitochondrial functional changes in htau mice are specific to synaptic mitochondria, non-synaptic and synaptic mitochondria were isolated from htau and WT male mice at 5- and 8-months of age and OCR driven by complex II (succinate as substrate) were measured utilizing the coupling assay (Rogers et al., 2011). Non-synaptic mitochondria from htau mice did not show alterations in respiration compared to WT mice at either age examined (Figures 3A,B). Synaptic mitochondria isolated from htau mice at 8- (but not 5-) months of age exhibit a significant increase in the rate of complex II driven state 2 (basal), state 3 (ADP-stimulated), and state 3u (maximum uncoupled) respiration compared to those from age-matched WT mice **(Figures 3C,D)**. Accumulation of hyperphosphorylated tau begins by 6 months in htau mice (Andorfer et al., 2003); thus, mitochondria from the synapse appear particularly sensitive to pathologic tau accumulation, and exhibit synaptic mitochondrial functional changes reported during normal aging (Stauch et al., 2014a).

### Quantitative Mitochondrial Proteomics Reveals Distinct Metabolic Pathways Are Altered in Synaptic Versus Non-synaptic Mitochondria From Htau Mice

**FIGURE 4:**
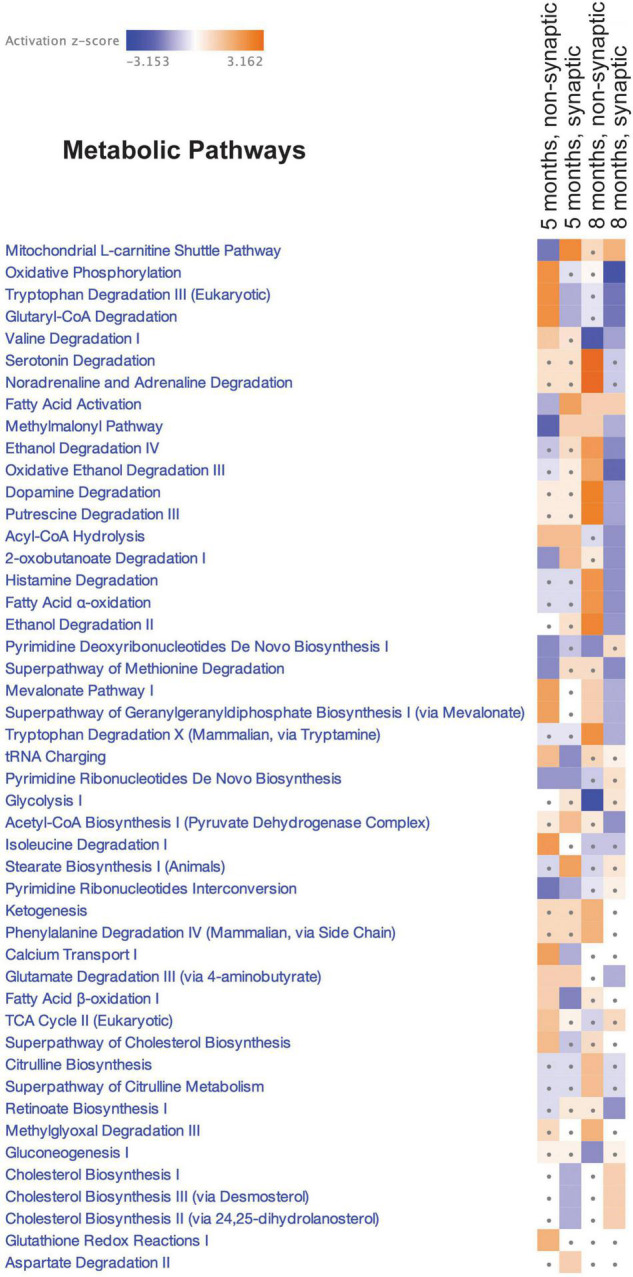
Canonical pathway enrichment analysis. Heatmap representing enriched metabolic pathways produced by IPA canonical pathway analysis of the SWATH-MS Log_2_ htau/WT protein expression data generated from an all-vs.-all comparison of the mitochondrial populations. Score filter p-value cutoff = 1.3 (log10) and z-score cutoff = 1 (absolute value). Blue blocks and red blocks represent inhibited and activated metabolic pathways, respectively. Gray dots represent metabolic pathways that did not achieve significance in the given experimental comparison.

Synaptic accumulation of tau has been reported to alter synaptic bioenergetics; however, the precise mechanisms remain poorly described (reviewed in Pérez et al., 2018a). We next sought to identify potential mechanisms that may, in part, contribute to the observed alterations in synaptic mitochondrial bioenergetics in htau mice. Synaptic and non-synaptic mitochondria were isolated from 5- and 8-month-old htau and WT male mice, and the proteome was analyzed using the quantitative mass spectrometry-based technique SWATH-MS (Gillet et al., 2012). In total, 1,313 mitochondrial proteins were identified (annotated using MitoMiner) and the complete list of these proteins with quantitative values is provided in Supplementary Table 1. Comparison Analysis was performed using Ingenuity Pathway Analysis (IPA) Software to visualize the SWATH-MS results to identify global proteomic similarities and differences between the mitochondrial populations. Heat maps were generated for Canonical Pathways Analysis (Metabolic Pathways, Figure 4). The Metabolic Pathways analysis revealed differential alterations in multiple pathways, in particular “Oxidative phosphorylation” had the strongest negative activation z-score (−2.566) for synaptic mitochondria from htau mice at 8 months predicting inhibition of this pathway, supporting our finding of impaired mitochondrial maximal respiration and spare respiratory capacity in synaptosomes. While the activation z-scores for this pathway are not considered significant for non-synaptic mitochondria at 8 months or synaptic mitochondria at 5 months, a positive activation z-score (2.321) for non-synaptic mitochondria at 5 months predicts activation of the “Oxidative phosphorylation” in htau mice. Additionally, pathways related to cholesterol metabolism (Cholesterol Biosynthesis I, II (via 24,25-dihydrolanosterol), and III (via Desmosterol) displayed negative activation z-scores in synaptic mitochondria of htau mice aged 5 months that were conversely positive at 8 months and unchanged in non-synaptic pools at either age (Figure 4). The “Superpathway of Cholesterol Biosynthesis” was also predicted to have negative activation z-score in 5-month-old htau synaptic mitochondria albeit not significant.

**FIGURE 5:**
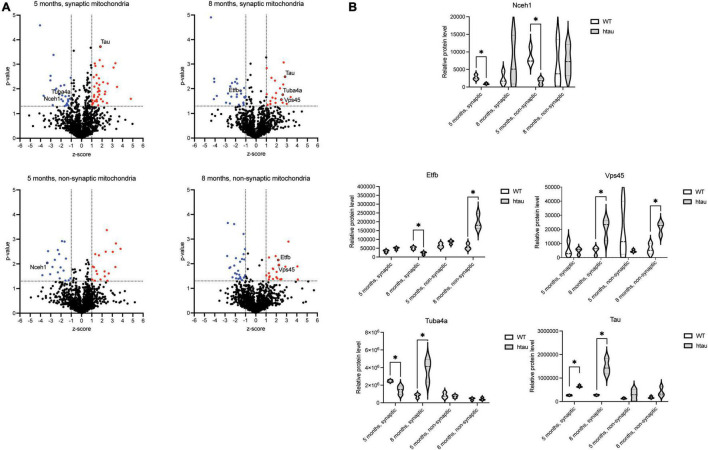
Identification of differentially expressed mitochondrial proteins. **(A)** The distribution of *p*-values (−log10) and z-scores (log_2_) in 1,313 quantified mitochondrial proteins between the htau and WT mice. A total of 65 (5 months, synaptic), 47 (8 months, synaptic), 38 (5 months, non-synaptic), and 49 (8 months, non-synaptic) proteins were selected as differentially expressed, which exhibited a *p*-value < 0.05 and a z-score > 1 standard deviation (highlighted in red). **(B)** Quantification of relative expression of indicated differentially expressed proteins as determined by SWATH-MS analysis. Differential expression (*p*-value < 0.05 and z-score > 1 standard deviation (annotated by a*).

To uncover which individual proteins were differentially expressed (DE) in mitochondria isolated from htau as compared to age-matched WT mice, a ratio threshold (log_2_ htau/WT) was determined in addition to a *p*-value threshold. The ratio threshold was determined from the normal distribution fit using 1 standard deviation, where the absolute value of the z-score [normalized log_2_ ratios (htau/WT)] had to be superior to 1.0 (Figure 5A). To obtain *p*-values, the protein expression values for the biological replicates were analyzed using a Student’s *t*-test analysis, using *p* < 0.05 (uncorrected) for the threshold. Combining these thresholds, we obtained lists of 38 (19 downregulated and 19 upregulated, 5-months) and 49 (27 downregulated and 22 upregulated, 8-months) DE proteins in non-synaptic mitochondria samples, and 65 (27 downregulated and 38 upregulated, 5-months) and 47 (27 downregulated and 20 upregulated) 8-months) DE proteins in synaptic mitochondria samples (Figure 5A and Supplementary Tables 2A–D). Despite the IPA analysis pointing to “Oxidative phosphorylation” pathway inactivation based on global proteomic changes, only 1 subunit, Ndufv3 (NADH dehydrogenase [ubiquinone] flavoprotein 3), an accessory subunit of complex I was found to be significantly changed in synaptic mitochondria from htau mice at 8 months. Notably, there was minimal overlap of DE proteins between groups, with only 1 DE protein (Nceh1, neutral cholesterol ester hydrolase 1) in common between non-synaptic and synaptic mitochondria at 5-months, 2 DE proteins (Etfb, electron transfer flavoprotein subunit beta and Vps45, vacuolar protein sorting 45 homolog) in common between non-synaptic and synaptic mitochondria at 8-months, 0 DE proteins in common between non-synaptic mitochondria at 5- and 8-months, and 2 DE proteins (Tuba4a, tubulin alpha 4a and Mapt, microtubule associated protein tau) in common between synaptic mitochondria at 5- and 8-months (Figures 5A,B).

Bioinformatic analysis of the DE proteins using DAVID functional annotation tool revealed several GO biological process terms that were altered according to our proteomic results (Table 1 and Supplementary Tables 3A–D). The top two enriched terms associated with the DE lists based on the percentage of involved genes/total genes revealed “oxidation-reduction process” and “apoptotic process” (5 months, synaptic), “microtubule-based process” (8 months, synaptic), “oxidation-reduction process” and “translation” (5 months, non-synaptic), and “transport” and “protein transport” (8 months, non-synaptic) were altered in htau as compared to WT mice. Taken together, our synaptic mitochondrial protein proteomic analysis in htau mice implicate a role for tau pathology in bioenergetic impairment, transport-based processes, and cholesterol metabolism.

**TABLE 1:** Enrichment of GO biological process terms for differentially expressed proteins in htau mice.

Mitochondrial pool	Biological process term	%	*p*-Value	Genes
5 months, synaptic	oxidation-reduction process	15.4	2.80E-04	Bdh1, ND5, Acadm, Akr7a5, Aifm1, Chchd4, Ethe1, Gpd1, Moab, Ptgs1
	apoptotic process	10.8	9.70E-03	Dnaja3, Aifm1, Fam162a, Fis1, Mapt, Sgpl1, Trpv2
8 months, synaptic	microtubule-based process	6.4	4.50E-03	Tuba4a, Tubb3, Tubb4a
5 months, non-synaptic	oxidation-reduction process	15.8	1.00E-02	Ndufb8, Cat, Dhrs4, Hsd17b12, Mthfd1, Pcyox1
	translation	13.2	8.00E-03	Eif4gl, Mrpl17, Mrpl55, Rpl12, Slc25a51
8 months, non-synaptic	transport	22.4	1.10E-02	Atp5e, Atp1b3, Cadps, Etfb, Eif5a, Gosr2, Lrpprc, Mtx2, Nup155, Slc6a6, Vps45
	protein transport	12.2	1.40E-02	Cadps, Eif5a, Gosr2, Mtx2, Nup155, Vps45

*Differentially expressed proteins in mitochondria isolated from htau mice were analyzed using DAVID to identify biological processes affected by human tau expression.*

### Synaptic Tau Accumulation Does Not Alter Mitochondrial Content in Synaptosomes

**FIGURE 6:**
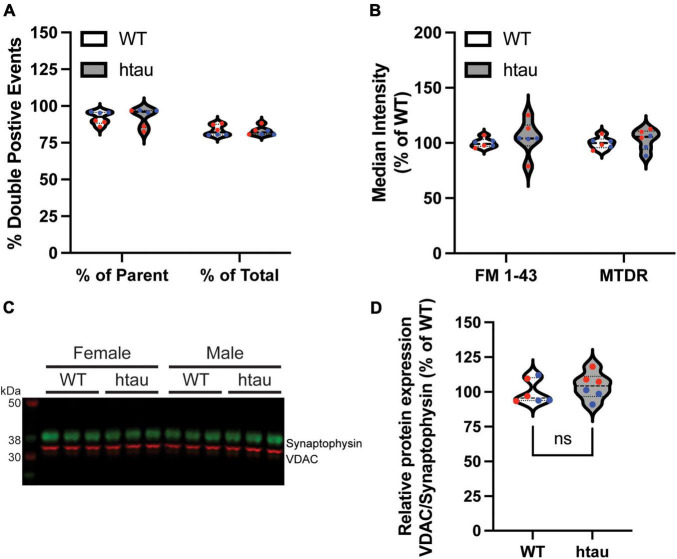
Mitochondrial content of synaptosomes is unaffected by human tau expression. Synaptosomes were isolated by discontinuous Percoll gradient from WT and htau mice at 8-months of age, doubled-labeled with FM 1-43 (a marker of synaptic membranes) and MitoTracker Deep Red (MTDR) and analyzed by flow cytometry. **(A)** Percent of double-labeled synaptosomes. **(B)** Median fluorescence intensity of each label. **(C)** Immunoblot analysis of isolated synaptosomes comparing Vdac1 (mitochondrial marker, green) and synaptophysin (synaptosome marker; red) and **(D)** densitometric quantification. Individual biological replicate points are included in violin plots male values in blue and female in red. Two-way ANOVA was used to assess statistical significance for flow experiments (*n* = 6 per genotype, males and females combined) and two-tailed unpaired Student’s T-Test for immunoblot (*n* = 6 per genotype, males and females combined). ns, non-significant.

Since our proteomics identified “microtubule-based process” as a top GO biological process term based on the list of DE proteins in synaptic mitochondria at 8 months, one possible explanation for our observed bioenergetic alterations in synaptic terminals of htau mice could be altered mitochondrial content. To assess this, we used flow cytometry to compare the mitochondrial content of synaptosomes between WT and htau mice. Isolated synaptosomes were incubated with MitoTracker Deep Red (MTDR) to label functional mitochondria and FM 1-43 to label synaptosomal membranes, and the percentage of double-labeled gate events were compared (Figure 6). For each sample approximately 120,000 events were captured and subsequently gated by size using forward and side scatter yielding a parent population (P1) of approximately 100,000 events. Double-labeled synaptosomes comprised approximately 80% of total population and 96% of the P1 population of synaptosomes isolated from both WT and htau animals (Figure 6A). Mean fluorescence intensity of FM 1-43 and MTDR (Figure 6B) did not differ significantly between WT or htau synaptosomes suggesting the mitochondrial biomass of synaptosomes did not change as consequence of human tau expression. Representative gating strategy and population plots are supplied in Supplementary Figure 2. To further confirm, we performed immunoblotting on isolated synaptosomes and compared the relative expression of voltage-dependent anion channel 1 (Vdac1), a commonly used mitochondrial loading control, which was found to be unaltered in our proteomics data, to that of synaptophysin, a well-established marker of synaptic terminals (Figure 6C). Ratiometric quantitation of our results revealed comparable levels of Vdac1 expression in WT and htau synaptosomes that was consistent between sexes. To eliminate the possibility that fluctuation of synaptophysin expression impacted relative quantitation, we also quantified Vdac1 as a function of total protein loading achieved with Coomassie bright blue staining (Supplementary Figure 3A). Like normalization to synaptophysin, we did not observe a significant difference in the relative expression of Vdac1 (Supplementary Figure 3B), and the two methods were well correlated (Supplementary Figure 3C). Although we cannot rule out contributions of mitochondrial biosynthesis in synaptic terminals, taken together, our data suggest that tau accumulation does not impact steady-state mitochondrial biomass in synaptic terminals.

### No Alterations in mtDNA Mutation Load or Copy Number at the Synapse in Htau Mice

**FIGURE 7:**
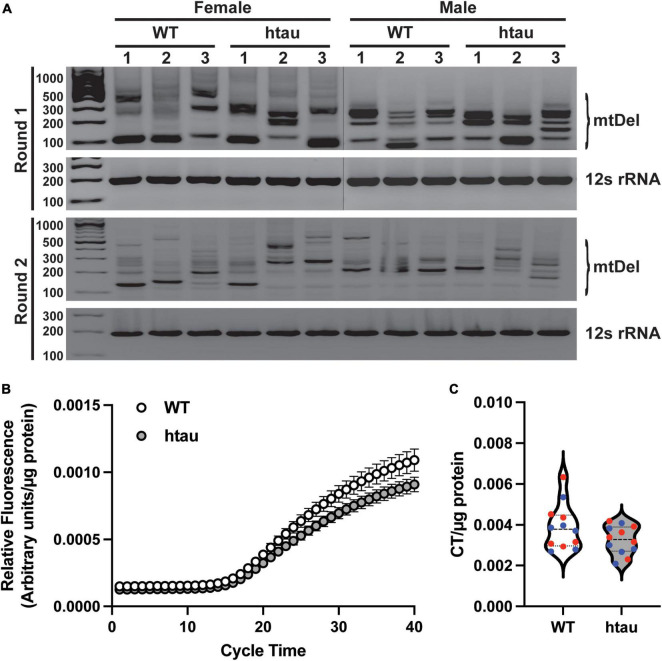
Tau accumulation does not promote mitochondrial genome instability. mtDNA was isolated from purified synaptosomes and analyzed by nested PCR to identify deletions and real-time quantitative PCR to determine mtDNA copy number. **(A)** Representative agarose gel images of nested PCR, deletion events are indicated by the presence of banding between ∼900 and ∼200 bp. The 12S region was amplified in parallel as loading control and to represent a region of mtDNA that is not susceptible to deletions. **(B)** Amplification curves of mtDNA copy number obtained by RT-qPCR with a TaqMan probe designed against 3155-3309 of the mouse mitochondrial genome. **(C)** Threshold cycle time values for mtDNA copy number. In B and C values were normalized to mg of synaptosome input for DNA isolation. Statistical significance was tested by two-tailed unpaired Student’s T-Test [*n* = 6 per sex and genotype; males (blue) and females (red) combined].

Our proteomics revealed that two proteins involved in mtDNA quality control, Tfam (transcription factor A) and Dguok (deoxyguanosine kinase) were significantly upregulated in synaptic mitochondria from htau mice at 8 months. Tfam is a key mitochondrial transcription factor and Dguok is a mitochondrial deoxyguanosine kinase involved in the mitochondrial nucleotide synthesis, thus we reasoned mtDNA aberrations could contribute to the changes in synaptic bioenergetics. We therefore assessed mtDNA to determine if tau accumulation conferred susceptibility to mtDNA instability or content at the synapse. First we sought to determine if tau accumulation promoted reduced mtDNA fidelity using a nested PCR assay that highlights deletions in a region of mtDNA known for lability (Brossas et al., 1994; Samuels et al., 2004; Ameur et al., 2011). We observed several bands representing mtDNA deletions in synaptosomes of both htau and WT mice, suggesting tau does not promote mtDNA instability (Figure 7A). Interestingly, deletion banding patterns were more pronounced in female htau mice with respect to WT, than in males (Figure 7A). Mitochondrial maintenance is a dynamic process comprised of biogenesis, turnover, fission, and fusion (Zhao, 2002; Pakos-Zebrucka, 2016; Eckl et al., 2021). Importantly, under stressed conditions the fusion process alters the degree of mitochondrial network connectivity by joining mitochondria in order to complement damaged mitochondria with healthy ones, a process that may alter mtDNA content (Chen et al., 2010). To determine if tau expression results in altered mtDNA content we used RT-qPCR to measure mtDNA copy number in synaptosomes (Figure 7B). We did not observe a significant difference in mtDNA copy number between WT or htau animals regardless of sex (Figure 7C). Notably, this is in support of our flow cytometry and immunoblot analyses of synaptosomes that suggested tau expression does not alter mitochondrial content of synapses (Figure 6).

### Accumulation of Pathologic Tau in Association With Synaptic Mitochondria

**FIGURE 8:**
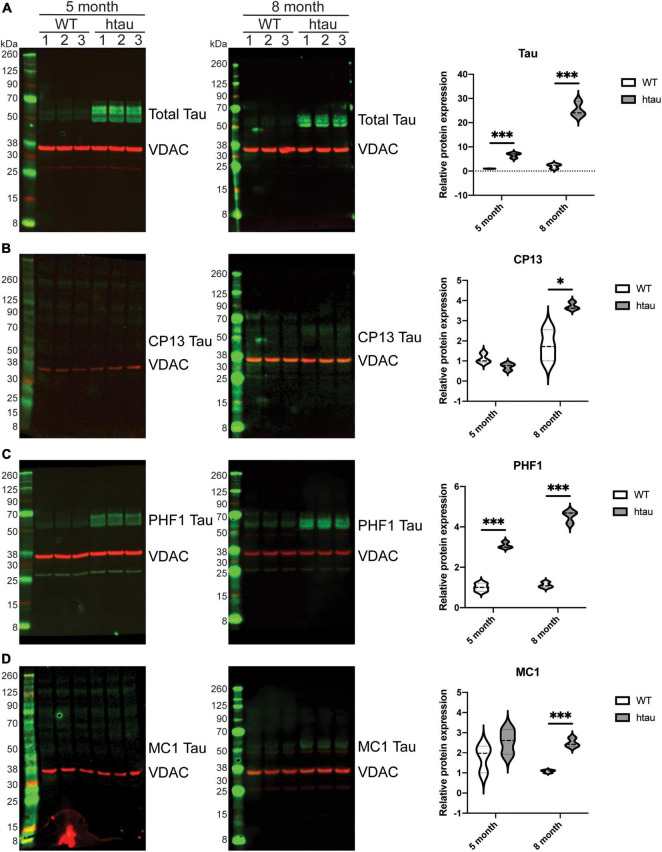
Total and phosphorylated tau is elevated in synaptic mitochondrial isolates from htau mice. Synaptic mitochondria were isolated by discontinuous Percoll gradient from 5- and 8-month-old WT or htau mice, solubilized with SDS buffer and analyzed by immunoblot for **(A)** total and **(B–D)** phosphoforms of tau (green). Quantification of immunoblots was done by densitometry, normalizing the indicated tau form to the mitochondrial marker Vdac1 (red). Statistical significance was determined by multiple T-Tests using the two-stage setup method (Benjamini, Krieger, and Yekutieli) (*p < 0.05, ^***^p < 0.001, *n* = 3 per genotype, males).

Our SWATH-MS analysis revealed that tau itself was significantly up-regulated in synaptic (but not non-synaptic) mitochondria isolated from htau mice at 5- and 8-months of age. We used immunoblotting to confirm the presence of tau associated with isolated synaptic mitochondria (Figure 8). Congruent with our SWATH-MS analysis (Figure 5B) we observed a significant increase in the amount of total tau protein present in synaptic mitochondria isolated from htau mice at both 5- and 8-months of age compared with WT mice, which was more pronounced at 8- compared to 5-months of age (Figure 8A). We also detected CP13 (Duff et al., 2000; Lewis et al., 2000; Koss et al., 2018) and PHF1 (Greenberg et al., 1992; Otvos et al., 1994) positive immunoreactivity in isolated synaptic mitochondria, which specifically detect abnormal phosphorylated residues S202 (Figure 8B) and S396/404 (Figure 8C), respectively. In synaptic mitochondria we observed low but comparable levels of CP13 immunoreactivity between 5-month-old WT and htau mice, however, at 8-months we detected a significant increase of CP13 signal in synaptic mitochondria from htau mice (Figure 8B). In contrast to CP13, we detected a significant increase in PHF1 signal at both 5- and 8-months in synaptic mitochondria from htau mice as compared to WT, and this exaggerated at 8-months (Figure 8C). In addition to pathologic hyperphosphorylation, tau also changes its conformation during disease progression (Jicha et al., 1997), which finally culminates in aggregation and formation of tangles. These early conformational changes can be monitored by probing with the antibody MC1, which is specific for a pathologic conformation of tau as it recognizes NFTs (Jicha et al., 1997). In synaptic mitochondria from 5-month-old htau mice there was a trending albeit insignificant increase in MC1 signal that achieved significance at 8-months (Figure 8D). Interestingly, a significant increase in the detection of PHF1 tau was observed at both 5- and 8-months timepoints, suggesting increased phosphorylation at S396/404 may be an earlier event in the progression of AD and related tauopathies (Figure 8C).

### Tau Localizes to the Synaptic Mitochondrial Outer Membrane

**FIGURE 9:**
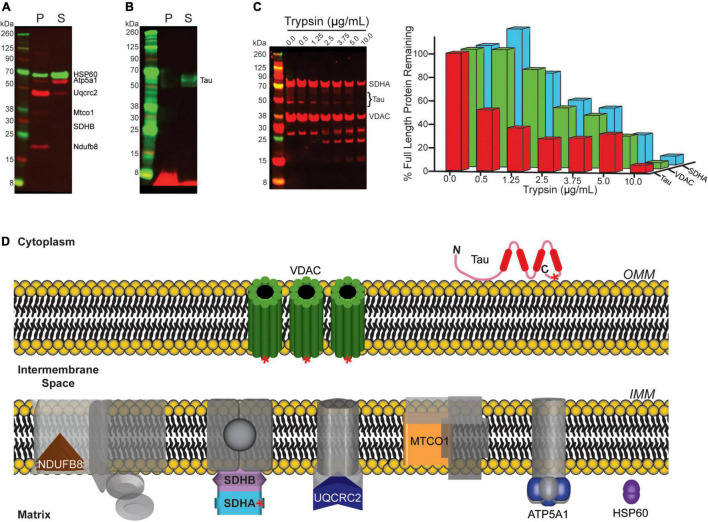
Mitochondrial associated tau is not membrane integrated. **(A,B)** Mitochondria isolated from 8-month-old htau mice were incubated on ice in a sodium carbonate extraction buffer and fractionated by ultracentrifugation to differentiate membrane associated from membrane-integrated proteins. The resulting fractions were assessed by immunoblot for the indicated proteins (P = pellet/insoluble (membrane-integrated), S = soluble (membrane associated). **(A)** Blot for Hsp60 and OxPhos as representative controls for the assay. **(B)** Blot of total tau. **(C)** Synaptic mitochondria isolated from 8-month-old htau mice were digested with the indicated concentration of trypsin on ice for 30 min, and then solubilized with SDS buffer and analyzed by immunoblot for total Tau, Sdha, and Vdac1 to determine the extent of protease digestion. **(D)** Diagram illustrating mitochondrial sub-localization of the proteins probed for in **(A–C)**. Red asterisks on Vdac1, Tau, and Sdha represent relative epitope location for the antibodies used in immunoblotting following protease treatment.

We next sought to determine the mechanism by which tau copurifies with synaptic mitochondria. To address this, we used sodium carbonate extraction, an established method to differentiate integral mitochondrial membrane proteins from membrane associated mitochondrial proteins. In this assay integral membrane proteins fractionate into an insoluble (pellet; “P”) fraction, while peripheral membrane (i.e., membrane associated) proteins segregate into the soluble (“S”) fraction. Synaptic mitochondria isolated from 8-month-old htau mice were extracted with sodium carbonate and the resulting fractions were probed by immunoblot (Figure 9). As controls, we probed for the OxPhos panel (Figure 9A, red) as well as heat-shock protein 60 (Hsp60) (Figure 9A, green). Except for ATP synthase F1 subunit alpha (Atp5a1), we primarily detected the proteins of the OxPhos panel in the insoluble fraction, as expected, since most are integral membrane proteins, also consistent with localization, we observed the majority of Hsp60 in the soluble fraction. Finally, we observed tau protein segregating almost entirely into the soluble fraction (Figure 9B). Our results suggest that tau associated with synaptic mitochondria is not inserted into the mitochondrial membrane, but likely associated via protein-protein interaction. To further support our sodium carbonate extraction results, we employed a protease accessibility assay to determine the rate at which tau was degraded compared to two mitochondrial proteins with known localization. We reasoned that if tau was associated with the mitochondrial outer membrane via protein-protein interactions it would be degraded at lower concentrations of protease than either integral membrane or mitochondrial matrix proteins. Isolated synaptic mitochondria from 8-month-old htau mice were digested with trypsin and immunoblotted for tau, Vdac1 (integral outer membrane protein), and succinate dehydrogenase complex subunit A (Sdha, matrix protein) (Figure 9C). We observed a reduced percentage of remaining full length tau protein at lower trypsin concentration than either Sdha or Vdac1, suggesting mitochondrially associated tau is more accessible to proteolytic action. This is consistent with the location of the epitopes recognized by the antibodies used for detection (Figure 9D). Taken together with our sodium carbonate extraction results, this suggests that tau is present on the mitochondrial outer membrane and this interaction is most likely mediated by protein-protein interactions.

## Discussion

Our results revealed synaptic mitochondrial changes caused by expression of non-mutant human tau in mice *in vivo*. Our analyses suggest functional disruption of the synaptic mitochondrial response to increased energy demands (i.e., neurotransmission) in the presence of tau protein exhibiting phosphorylation at AD-associated residues accumulating in synaptosomes in htau mice, which is accompanied by tau association with synaptic mitochondria that exhibit an aged bioenergetic phenotype. These abnormalities exist in the absence of changes to synaptosomal mitochondrial content and consistent with this basal mitochondrial respiration and ATP generation remained unaltered in htau mice. Given our findings that tau preferentially associates with synaptic mitochondria and that non-synaptic mitochondria remain functionally intact, the bioenergetic consequences of synaptic mitochondria-tau interactions may have important contributions to synaptic dysfunctions in AD.

Synaptic accumulation of tau has been observed in human AD samples. One study from 2008, reported the accumulation of tau protein in synaptosomes isolated from brains perimortem, where it colocalized with amyloid (Fein et al., 2008). Furthermore, fluorescent imaging demonstrated hyperphosphorylated tau primarily localizes in neurites of intact neurons (Fein et al., 2008). Hyperphosphorylated and detergent-stable tau oligomers were detected in synaptosomes of AD patients as well as the 3xTG.AD mouse model (Henkins et al., 2012). In another study of postmortem dementia patient samples, synaptic accumulation of both total and hyperphosphorylated tau was reported in conjunction with ubiquitin-proteosome dysfunction (Tai et al., 2012). Subsequent studies have reported an increased presence of tau (normal and pathological) in isolated pre- and post-synaptic terminals from frozen human AD patient samples (Tai et al., 2014; Gylys and Bilousova, 2017). Furthermore, accumulation of total and PHF tau was reported in synaptosomes of AD brains and increased with Braak stage (DeVos et al., 2018). Our results reveal that, like human AD patients, an increased presence of tau (total and phosphoforms) is observed in synaptosomes from htau mice. In particular, we found significantly elevated levels of several phosphoforms of tau, including pS199, pT231, and pS396. Our findings lend support to the role of non-mutant human tau in synaptic dysfunction in AD. However, a recent report using super-resolution microscopy of fixed synaptosomes from WT (C57BL/6) mice observed significant colocalization of non-mutant murine tau with synaptophysin (marking pre-synaptic terminals) (Bhattacharya et al., 2021). Interestingly, the authors noted tau also colocalized with the post-synaptic terminal marker protein post-synaptic density protein 95 (PSD-95), suggesting tau accumulates in both compartments of the synapse (Bhattacharya et al., 2021). Subcellular fractionation analysis from mutant tau^*P*301*L*^ mice showed that both murine tau and the human tau^*P*301*L*^ were present in the synaptosomal fraction; however, tau^*P*301*L*^ was preferentially distributed in the synaptosomal fraction while murine endogenous tau was more distributed in the cytosolic fraction (Sahara et al., 2014). Interestingly, human-specific tau with phosphorylation at pS199 and pS396 was observed in the synaptosomal fraction of tau^*P*301*L*^ mice (Sahara et al., 2014). A study in Drosophila reported tau localization and accumulation in pre-synaptic terminals and similarly to Sahara et al. (2014), it was demonstrated to associate with the pre-synaptic membrane (Zhou et al., 2017). Thus, comparative studies into the synaptosomal distribution of tau protein and the tau phosphorylation profile within synaptosomes between samples from AD patients and AD mouse models will be valuable to understand tau contributions to synaptic dysfunction.

Unfortunately, bioenergetic assessments of synaptosomes isolated from postmortem human tissue are inhibited by the detrimental effects of cryopreservation on synaptosome structural and functional integrity. Previously, bioenergetic assessments of synaptosomes from commonly used mouse models with AD-like phenotypes revealed no consistent bioenergetic deficiencies from three models [J20 age 6 months, Tg2576 age 16 months, APP/PS age 9 months (Choi et al., 2012)], suggesting the intrinsic energetic capacity of synaptosomes were maintained in these symptomatic AD mouse models. Here, we assessed synaptosomes from htau mice aged 8 months, and uncovered bioenergetic deficits in mitochondrial maximal and spare respiratory capacity, coinciding with our findings of synaptosomal tau accumulation. Of note, no changes in basal mitochondrial respiration nor basal ATP production were found. These findings suggest synaptic accumulation of tau induces an impairment in the ability of synaptic mitochondria to respond to increased energy demands, a function necessary for neurotransmission. Despite findings *in vitro* that tau accumulation impairs both antero- and retro-grade axonal trafficking of molecular cargos, including mitochondria (Iqbal et al., 1998; Stamer et al., 2002; Kanaan et al., 2011; Zhang et al., 2012; Tai et al., 2014; Li et al., 2016; Sheng and Cai, 2016; Lacovich et al., 2017; Zhou et al., 2017), we found that the mitochondrial content of synaptosomes did not differ between WT and htau mice. Similarly, the volume fraction of mitochondria does not differ between synaptosomes from WT and J20 mice (Choi et al., 2012). In absence of alterations in mitochondrial biomass, another mediator of bioenergetics is mitochondrial genome fidelity, in which, alterations in mtDNA sequence or copy number have been reported in several neurological disorders and are reported to impact mitochondrial function (Samuels et al., 2004; Lin and Beal, 2006; Chen et al., 2010; Manczak et al., 2011; Reddy et al., 2011; Xie et al., 2015). Our results suggest that tau accumulation did not promote increased mtDNA instability as deletion profiles were comparable between WT and htau mice, nor was mtDNA copy number significantly altered in synaptosomes. In line with this our proteomic analysis of synaptic mitochondria revealed that expression levels of the mtDNA encoded proteins remained unaffected in htau mice, and only identified reduced expression of one complex I accessory protein, Ndufv3. As this subunit is not part of the complex I core assembly, it seems unlikely this is a major contributor to our observed bioenergetic alterations (Bourges et al., 2004). However, IPA revealed the activation score for oxidative phosphorylation in synaptic mitochondria of 8-month htau mice was decreased, suggesting global proteomic changes in this pathway may act in concert to impart reduced function in response to increased energy demands. Recently, a study investigating global proteomic changes reported altered expression of a number of mitochondrial proteins in AD patient brain regions in early and late stages of disease (Mendonça et al., 2019). Notably, expression of several mitochondrial proteins was observed to decrease, specifically in the entorhinal and parahippocampal cortices, two regions known to be affected by AD (Chen et al., 2014; Criscuolo et al., 2017; Mendonça et al., 2019). Furthermore, the study reported a decrease in the expression of mtDNA encoded complex I subunits, Mtnd4 and Mtnd1, in late stage AD (Mendonça et al., 2019). Mtnd4 is principle component of the minimal assembly core for complex I, and is critical for proper ETC function (Bourges et al., 2004). Although, we did not observe a significant difference in the expression of either Mtnd1 or Mtnd4, this difference could be the result of the stage of disease studied, mitochondrial pool differences, or intrinsic differences between humans and mice neurophysiology.

In isolated mitochondria, while non-synaptic mitochondria remained functionally intact, we found a significant elevation in both basal and maximal complex II-mediated respiration in synaptic mitochondria from 8-month-old (but not 5-month-old) htau mice as compared to WT controls. These changes are characteristic of an aged synaptic mitochondrial bioenergetic phenotype, which we previously reported in WT mice (Stauch et al., 2014a). Although elevated state 3 and state 3u respiration of isolated synaptic mitochondria seems to contrast the finding of impaired maximal and spare respiratory capacity of synaptic mitochondria within synaptosomes, it is important to point out that the experimental conditions differ between the assays performed [complex II coupling assay (isolated mitochondria) vs. MST (synaptosomes)], which likely account for this apparent discrepancy. Firstly, the starting substrate conditions differ, in the MST assay glucose and pyruvate are provided allowing for pyruvate oxidation to form acetyl coenzyme A and NADH, which are then oxidized by the tricarboxylic acid (TCA) cycle and complex I, respectively, for energy production. Further, in a series of enzymatic reactions, the TCA cycle generates the reducing equivalents NADH and FADH2, which are then oxidized by complex I and II, respectively. Thus, impairments observed in the MST assay could result from deficits in glycolysis (glucose converted to pyruvate releasing NADH and ATP), pyruvate oxidation, TCA cycle reactions, as well as complex I and II function. In contrast, in the complex II coupling assay succinate, which feeds directly into complex II, is provided in the presence of the complex I inhibitor rotenone, preventing detection of functional deficits in complex I, and bypassing the aforementioned pathways. Of note, we observed similar age-dependent complex II changes in the Pink1 KO rat model of Parkinson’s disease (PD) (Stauch et al., 2016), which in striatal synaptic mitochondria was accompanied by decreased complex I respiration. This apparent compensation, increased complex II activity in the presence of decreased complex I activity is reminiscent of the “inverse Warburg effect” where dysfunctional neurons increase OxPhos activity, which has been described in AD (Demetrius and Simon, 2012) and may in part explain the enhanced complex II state 3 and state 3u respiration we observed. While we did not directly interrogate complex I function in htau mice, a recent *in vitro* study reported expression of human tau in primary neuronal cultures impaired complex I activity by increasing mitofusin expression (Li et al., 2016). Li et al. (2016) also reported increased mitofusin expression and decreased complex I driven oxygen consumption in hippocampal extracts of htau mice. Another study reported that genetic ablation of tau expression improved hippocampal mitochondrial function despite reducing expression of proteins of the electron transport chain (ETC) (Jara et al., 2018). Furthermore, studies investigating pathological mutant forms of tau have similarly reported alterations is bioenergetics. One such study by David et al. (2005), demonstrated expression of human mutant tau^*P*301*L*^ in mice impaired respiratory control ratio and ATP production from cerebral mitochondrial isolates.

Our proteomics analysis of synaptic mitochondrial pools revealed the only common protein to be upregulated in htau mice at both ages was tau itself, which was not significant in non-synaptic mitochondrial pools. Further, we found that tau associating with synaptic mitochondria included phosphoforms (pS202 and pS396/404) and misfolded forms (MC1) of the protein. Other studies have reported the association of tau protein with mitochondria in a number of systems (Rhein et al., 2009; Vossel et al., 2010; Zündorf and Reiser, 2011; Zhang et al., 2012; Hu et al., 2016; Li et al., 2016; Cieri et al., 2018). This association was determined in some cases to result from incorporation of tau into the outer mitochondrial membrane, yet in others the consequence of protein-protein interactions with outer mitochondrial membrane proteins, one such protein is Vdac1 (Atlante et al., 2008; Quintanilla et al., 2009; Amadoro et al., 2011; Reddy, 2013). In htau mice, we determined that tau associated with synaptic mitochondria is not inserted into the mitochondrial membrane, but likely associates via protein-protein interaction. In line with this notion, other studies have reported aberrant protein-protein interactions between hyperphosphorylated tau and regulators of mitochondrial homeostasis (reviewed in Mietelska-Porowska et al., 2014). One study investigating aberrant interactions of tau with the mitochondrial fission protein Drp1, reported increased mitochondrial fission and fission linked-GTPase activity from post-mortem AD patient samples and in the APP, APP/PS1, 3xTG.AD mouse models (Manczak et al., 2011; Manczak and Reddy, 2012). Indeed, other studies have demonstrated the accumulation of tau impaired mitophagy by protecting the mitochondrial membrane potential to prevent activation of the Pink1/Parkin pathway (Hu et al., 2016) or by physical sequestration of cytosolic Parkin preventing its recruitment to damaged mitochondria (Cummins et al., 2019). The combination of increased GTPase activity and mitochondrial fission resulted in a fragmented mitochondrial phenotype the authors suggested could be a contributor to synaptic bioenergetic deficits and subsequent synapse loss (Manczak et al., 2011; Manczak and Reddy, 2012). Future studies focusing on determining mechanistically how tau preferentially associates with synaptic mitochondria will be of significant interest.

Our proteomic analysis revealed differential expression of proteins that, in part, contribute to mitochondrial assembly, transport, and maintenance, including cholesterol metabolism. Altered cholesterol metabolism is reported in several neurodegenerative disorders and may represent a common feature of neurodegeneration (reviewed in Arenas et al., 2017). In AD, this is exemplified by the association between propensity for hypercholesterolemia and AD risk in carriers of the ε4 variant allele of apolipoprotein E (APOE4), of which is present in as many as 65% of sporadic and late-onset familial AD cases, and is the greatest risk factor in non-familial cases (Dass et al., 1985; Saunders et al., 1993; Lin-Lee et al., 2002). Notably, precise mechanisms by which the ε4 allele increases AD risk are currently controversial, however, the ε4 allele is associated with enhanced oxidative stress, more severe amyloid pathology (Holtzman et al., 2012), disruption of neuronal signaling, and—most relevant to our study—altered phosphorylation of tau and promotion of NFT formation (Posse De Chaves and Narayanaswami, 2008; Shi et al., 2017). The brain—the organ with the highest concentration of cholesterol—relies heavily on cholesterol metabolism to maintain functional cholesterol balance, as a lipid carrying protein that transports lipids and cholesterol between cells of the brain, APOE contributes to cholesterol metabolism (Shore et al., 1974; Dietschy and Turley, 2001; Posse De Chaves and Narayanaswami, 2008; Holtzman et al., 2012). Furthermore, the original discovery of APOE reported its association with cholesterol esters (CE), a metabolic byproduct of cholesterol metabolism elevated in AD brains (Shore et al., 1974; Hutter-Paier et al., 2004). In neurons, cholesterol metabolism is balanced by the conversion of excess cholesterol to CE by acyl-CoA:cholesterol acyltransferase (ACAT) and reverted by cholesterol ester hydrolases, such as neutral cholesterol ester hydrolase 1 (Nceh1) (Sakai et al., 2014; Feringa and van der Kant, 2021). Accumulation of CE occurs in several neurodegenerative disorders and enhances accumulation of amyloid plaques in AD (Hutter-Paier et al., 2004). In the mutant APP1 and tau^*P*301*L*^ mouse models of AD, reduced amyloid deposition and enhanced autophagy were reported following inhibition or knock-out of ACAT1 (Hutter-Paier et al., 2004; Shibuya et al., 2014, 2015). ACAT inhibitors are being explored pre-clinically for AD, however, while human trials are lacking differential expression of cholesterol ester hydrolases has been observed in AD samples and primary rat cortical neurons transduced with APP (Brown et al., 2004; Hutter-Paier et al., 2004). Suggesting cholesterol:CE imbalance may not result from enhanced CE synthesis, studies on multiple sclerosis suggest that excess CE results from the impaired hydrolysis of CE rather than increased synthesis (Fewster et al., 1970; Shah and Johnson, 1980). In line with this, in synaptic and non-synaptic mitochondrial pools of 5-month htau animals, we observed reduced expression of Nceh1 without any significant changes in expression of ACAT1. This may suggest impaired CE hydrolysis is an early feature in onset and progression of AD-like pathology in the htau model. Moreover, alterations to cholesterol balance can alter the composition of mitochondrially associated ER membranes impairing lysosomal and autophagic processes (Koga et al., 2010; Annunziata et al., 2018). Specifically, inhibiting synthesis of CE is linked to enhanced autophagy and may improve clearance of toxic proteins (such as hyperphosphorylated tau) before aggregation occurs (Shibuya et al., 2014). In addition to reduced expression of Nceh1, pathway analysis predicting upstream regulators also revealed alterations in cholesterol metabolic pathways, including downregulation of the “Superpathway of Cholesterol Biosynthesis” in non-synaptic htau mitochondrial samples at 5-months. Our observed accumulation of total and phosphoforms of tau may reflect impaired clearance mechanisms, however, our focused use of mitochondrial isolates makes this difficult to determine in our proteomics analysis.

## Conclusion

In the current study we have reported that phosphoforms of non-mutant human tau protein accumulate in synaptosomes and associate with synaptic mitochondria *in vivo*. While synaptosomal mitochondrial content and basal mitochondrial respiration/ATP generation remained unaltered in htau mice, maximal mitochondrial respiration was impaired, suggesting failure to respond to increased energy demands as would be necessary for proper neurotransmission. While our results indicate tau accumulation is detrimental to the function of synaptic mitochondria, the precise molecular mechanisms that govern preferential associations between tau and synaptic mitochondria remain elusive, and thus should be the subject of detailed future investigations.

## Data Availability Statement

The data presented in this study are deposited in the ProteomeXchange Consortium via the PRIDE partner repository, accession number PXD030950.

## Ethics Statement

The animal study was reviewed and approved by University of Nebraska Medical Center Institutional Animal Care and Use Committee.

## Author Contributions

KS, HF, and AT conceived and designed the experiments. KS, KE, AT, NR, EL, and JG performed the experiments. KS, KE, AT, JG, and ST analyzed the data. KS, HF, AT, JG, KE, NR, EL, and ST wrote the manuscript. All authors read and approved the final manuscript.

## Conflict of Interest

The authors declare that the research was conducted in the absence of any commercial or financial relationships that could be construed as a potential conflict of interest.

## Publisher’s Note

All claims expressed in this article are solely those of the authors and do not necessarily represent those of their affiliated organizations, or those of the publisher, the editors and the reviewers. Any product that may be evaluated in this article, or claim that may be made by its manufacturer, is not guaranteed or endorsed by the publisher.

## Footnotes

1
http://mitominer.mrc-mbu.cam.ac.uk/release-4.0/begin.do

## Supplementary Material

Supplementary Figure 1 Immunohistochemistry of hippocampal sections from 5- and 8-month-old htau mice confirm onset of histopathological hallmarks of AD. Hippocampal sections from 5- and 8-month-old htau mice were fixed, embedded, and stained for CP13 or PHF1 tau in the CA3 and CA1 substructures.

Supplementary Figure 2 Gating strategy and compensation controls for flow cytometric analysis of synaptosomes. Representative plots of unstained controls, individual control stains, and each synaptosome sample analyzed by flow cytometry are presented.

Supplementary Figure 3 Total protein stain confirms tau does not alter synaptic mitochondrial content. Synaptosomes isolated from 8-month-old WT or htau mice of either sex were assessed by flow cytometry and immunoblot for mitochondrial content (Vdac1 as surrogate; see Figure 6C), quantification of total protein (Coomassie bright blue) was performed to validate Figure 6C. **(A)** Image of Coomassie bright blue staining demonstrating equal sample loading. **(B)** Quantification of Vdac1 signal as a function of total protein signal. **(C)** Correlation plot of Vdac1 normalized to total protein and Vdac1 normalized to synaptophysin. Individual biological replicate points are included in violin plots male values in blue and female in red. Statistics were determined by two-tailed unpaired Students’ *t*-Test (*n* = 6 per genotype, males and females combined).

Supplementary Table 1 Expression levels of synaptic and non-synaptic mitochondrial proteins from 5- and 8-month-old htau and WT mice as determined by SWATH-MS and annotated by MitoMiner. Supplementary Table 1A contains proteins annotated as mitochondrial and Supplementary Table 1B contains non-mitochondrial proteins.

Supplementary Table 2 Differentially expressed synaptic and non-synaptic mitochondrial proteins identified by SWATH-MS at 5- and 8-months of age in htau and WT mice. Protein list of significantly altered synaptic mitochondrial proteins in htau compared to WT mice for 5 month, non-synaptic (Supplementary Table 2A); 8 month, non-synaptic (Supplementary Table 2B); 5-month, synaptic (Supplementary Table 2C); and 8-month, synaptic (Supplementary Table 2D).

Supplementary Table 3 DAVID enrichment analysis of mitochondrial proteins differentially expressed between WT and htau mice for 5 month, non-synaptic (Supplementary Table 3A); 8 month, non-synaptic (Supplementary Table 3B); 5 month, synaptic (Supplementary Table 3C); and 8 month, synaptic (Supplementary Table 3D).

## Abbreviations

AAV: adeno-associated virus
ACAT: acyl-CoA:cholesterol acyltransferase
AD: Alzheimer’s disease
ATP: adenosine triphosphate
Atp5a1: ATP synthase F1 subunit alpha
BH: Benjamini–Hochberg
BP: biological process
BSA: bovine serum albumin
CE: cholesterol ester
DE: differentially expressed
Dguok: deoxyguanosine kinase
Drp1: dynamin-related GTPase 1
ER: endoplasmic reticulum
ETC: electron transport chain
Etfb1: electron transfer flavoprotein beta-subunit
FDR: false discovery rate
Fmr1: fragile X mental retardation 1
FTD: frontotemporal dementia
GO: gene ontology
HSP60: heat-shock protein of 60 kDa
IB: incubation buffer
IDCR: ionic detergent compatibility reagent
IPA: ingenuity pathway analysis
KO: knock-out
Map: microtubule-associated protein
Mapt: microtubule-associated protein tau
MAS: mitochondrial assay solution
MES: 2-(N-morpholino)ethanesulfonic acid
MOPS: 3-morpholinopropane-1-sulfonic acid
MS: mass spectrometry
MSHE: mitochondrial isolation buffer (Mannitol sucrose, HEPES EGTA)
MT: microtubule
mtDNA: mitochondrial DNA
MTDR: MitoTracker Deep Red
Mtnd: mitochondrially encoded NADH dehydrogenase
Nceh1: neutral cholesterol ester hydrolase 1
Ndufv3: NADH dehydrogenase [ubiquinone] flavoprotein 3
NFT: neurofibrillary tangles
NS: non-synaptic
OxPhos: oxidative phosphorylation
P: pellet
PD: Parkinson’s Disease
PHF: paired helical fragment
PSD-95: post-synaptic density protein 95
ROS: reactive oxygen species
RT: room temp
S: soluble
SB: solubilization buffer
SDHA: succinate dehydrogenase complex subunit A
SRC: spare respiratory capacity
STRING: search tool for the retrieval of interacting genes/proteins
SWATH-MS: sequential window acquisition of all theoretical mass spectra
Syn: synaptic
Tfam, transcription factor A: mitochondrial
Tuba4a: tubulin alpha 4a
Vdac1: voltage dependent anion channel 1
Vps45: vacuolar sorting 45 homolog
WT: wild-type.
