# Supplementary figures and images for: Hyperphosphorylated Human Tau Accumulates at the Synapse, Localizing on Synaptic Mitochondrial Outer Membranes and Disrupting Respiration in a Mouse Model of Tauopathy
Source: Front Mol Neurosci. 2022 Mar 10;15:852368. doi: 10.3389/fnmol.2022.852368 (PMC8960727; doi:10.3389/fnmol.2022.852368)

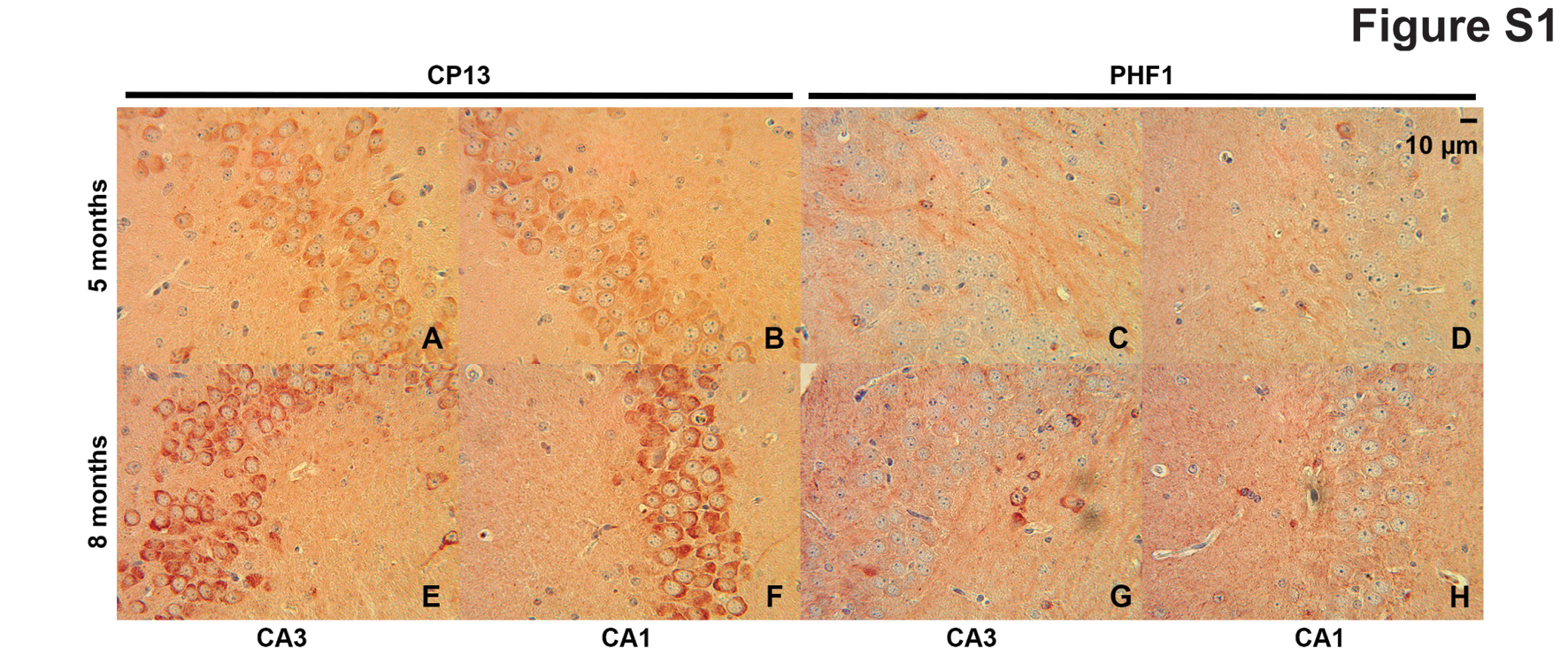

Supplement: Supplementary Figure 1 — Immunohistochemistry of hippocampal sections from 5- and 8-month-old htau mice confirm onset of histopathological hallmarks of AD. Hippocampal sections from 5- and 8-month-old htau mice were fixed, embedded, and stained for CP13 or PHF1 tau in the CA3 and CA1 substructures. [file Image_1.tif]

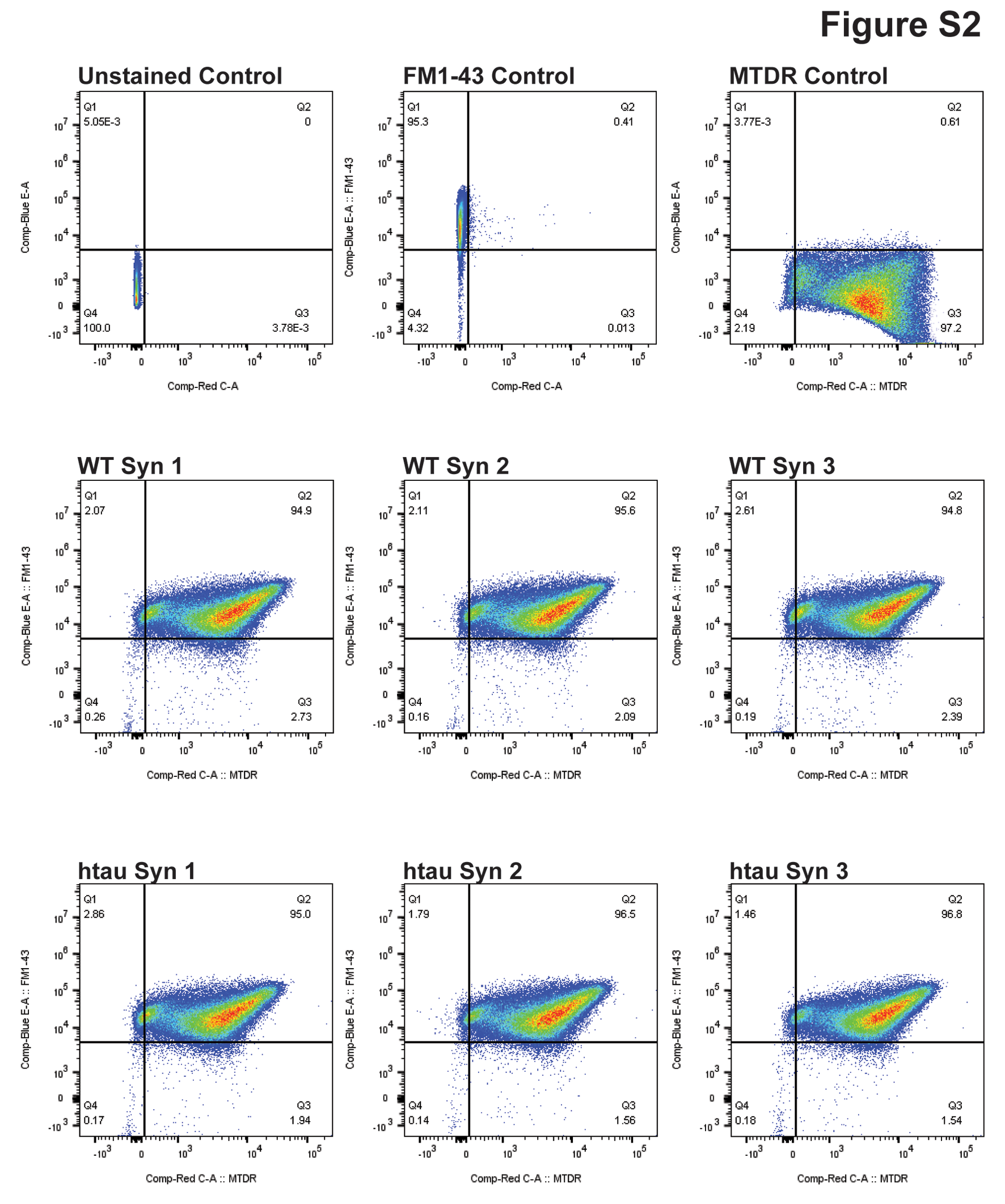

Supplement: Supplementary Figure 2 — Gating strategy and compensation controls for flow cytometric analysis of synaptosomes. Representative plots of unstained controls, individual control stains, and each synaptosome sample analyzed by flow cytometry are presented. [file Image_2.tif]

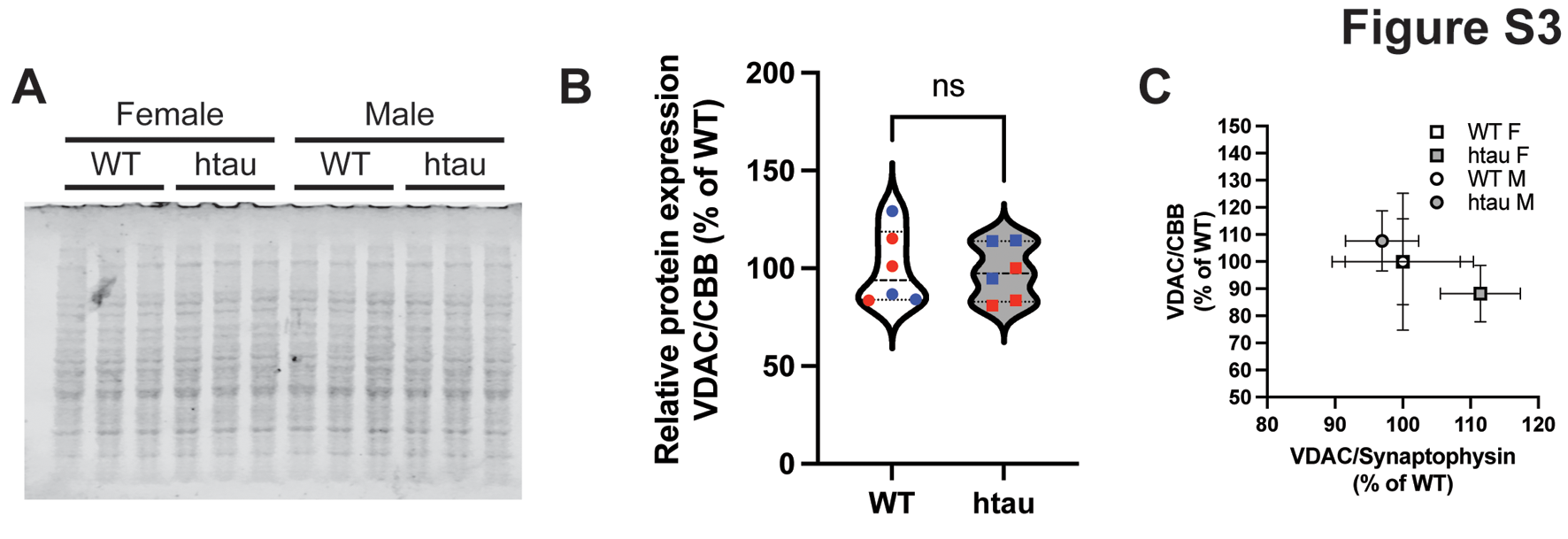

Supplement: Supplementary Figure 3 — Total protein stain confirms tau does not alter synaptic mitochondrial content. Synaptosomes isolated from 8-month-old WT or htau mice of either sex were assessed by flow cytometry and immunoblot for mitochondrial content (Vdac1 as surrogate; see Figure 6C), quantification of total protein (Coomassie bright blue) was performed to validate Figure 6C. (A) Image of Coomassie bright blue staining demonstrating equal sample loading. (B) Quantification of Vdac1 signal as a function of total protein signal. (C) Correlation plot of Vdac1 normalized to total protein and Vdac1 normalized to synaptophysin. Individual biological replicate points are included in violin plots male values in blue and female in red. Statistics were determined by two-tailed unpaired Students’ t-Test (n = 6 per genotype, males and females combined). [file Image_3.tif]
